# Gut microbiome and pregnancy complications: emerging evidence and mechanistic insights

**DOI:** 10.1080/19490976.2026.2661417

**Published:** 2026-04-23

**Authors:** Shijia Hu, Zelei Miao, Congmei Xiao, Yuanqing Fu, Jianqiong Zheng, Wensheng Hu, Ju-Sheng Zheng

**Affiliations:** a Affiliated Hangzhou First People's Hospital, School of Medicine, Westlake University, Hangzhou, China; b Westlake Laboratory of Life Sciences and Biomedicine, Hangzhou, China; c Department of Obstetrics and Gynecology, The Third Clinical Institute Affiliated to Wenzhou Medical University, Wenzhou People's Hospital, Wenzhou Maternal and Child Health Care Hospital, Wenzhou, China; d Department of Obstetrics, Women's Hospital, School of Medicine, Zhejiang University, Hangzhou, China; e Key Laboratory of Reproductive Genetics (Ministry of Education) and Women's Hospital, Institute of Medical Genetics and Development, School of Medicine, Zhejiang University, Hangzhou, China; f Research Center for Industries of the Future, School of Life Sciences, Westlake University, Hangzhou, China; g Zhejiang Key Laboratory of Multi-Omics in Infection and Immunity, School of Medicine, Westlake University, Hangzhou, China

**Keywords:** Maternal gut microbiome, pregnancy complications, short-chain fatty acids, inflammation, host–microbe interaction, sex hormones

## Abstract

The gut microbiome undergoes significant alterations during pregnancy. Perturbations in these microbial communities are increasingly associated with a range of pregnancy complications, including miscarriage, gestational diabetes mellitus, preeclampsia, preterm birth, and fetal growth restriction, among others. This review synthesizes current evidence on the dynamic changes in the maternal gut microecosystem including bacterial, fungal, and viral communities throughout gestation and examines its relationships with various pregnancy complications. We also summarize the underlying mechanisms driving these interactions, focusing on metabolic regulations involving short-chain fatty acids, bile acids, indoles, sex hormones, intestinal barrier integrity, and the modulation of maternal immune responses essential for fetal tolerance. Additionally, we discuss the lasting impact of the maternal microbiome on offspring health via vertical transmission and developmental programming. This review provides a conceptual framework that integrates mechanistic insights with clinical findings, with the goal of informing future research and supporting the development of microbiome-based interventions to improve maternal and neonatal health outcomes.

## 1. Introduction

The gut microbiome plays critical roles in regulating host metabolism and maintaining health status.^1^^,^^2^ Pregnancy represents a unique physiological state characterized by significant immunological, hormonal, and metabolic adaptations essential for fetal development and maternal well-being.^3^^,^^4^ These systemic changes, which extend to nearly every organ system, are also reflected in the remodeling of the maternal gut microbiome.^5^ Importantly, perturbations in the maternal gut microbiota have been increasingly associated with various pregnancy complications, including miscarriage, gestational diabetes mellitus (GDM), maternal obesity, intrahepatic cholestasis of pregnancy (ICP), preeclampsia (PE), preterm birth (PTB), fetal growth restriction (FGR), and mental health issues.^6^^,^^7^ Such complications pose substantial public health challenges, contributing to maternal and neonatal morbidity and mortality worldwide.^8^ These findings are reshaping our understanding of the gut microbiome as both a source of predictive biomarkers and a target for potential therapeutic strategies against pregnancy complications.

**Figure 1.**
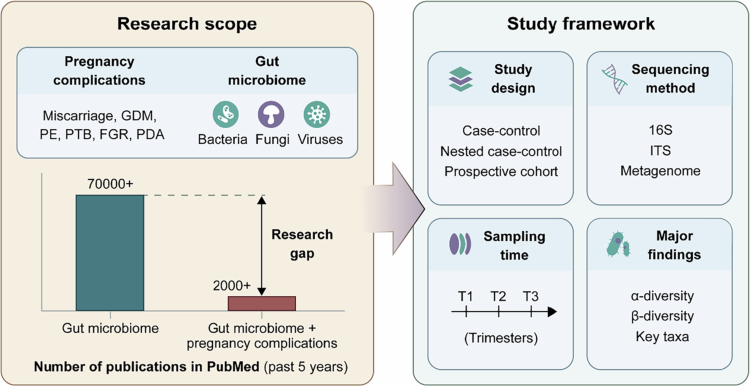
Current research scope and framework on maternal gut microbiome and pregnancy complications. Research scope overviews major pregnancy complications under investigation, including miscarriage, GDM, PE, PTB, FGR, and PDA, with the microbial domains studied (bacteria, fungi, and viruses). The number of publications underlines the relative under-investigation of the associations of gut microbiome with pregnancy complications compared with the gut microbiome. Research framework illustrates that current studies are predominantly case‒control or nested case‒control designs, with limited application of prospective cohort. Taxonomic profiling relies heavily on 16S rRNA and ITS sequencing, with emerging use of shotgun metagenomics to capture functional potentials. Sampling typically spans all three trimesters (T1, T2 and T3) to capture dynamic microbial shifts during gestation. Key analytical outputs include *α*- and *β*-diversity and identification of associated taxa. Abbreviations: GDM, gestational diabetes mellitus; PE, preeclampsia; PTB, preterm birth; FGR, fetal growth restriction; PDA, perinatal depression and anxiety; ITS, internal transcribed spacer; T1, the first trimester; T2, the second trimester; T3, the third trimester.

Although it has been shown that the maternal microbiome can influence health outcomes for both mother and child, the specific microbial signatures involved and the underlying mechanisms remain poorly understood. In recent years, reproductive and maternal health have gained renewed research attention, with increasing recognition of the complex interplay between the gut microbiota and female reproductive physiology.^9^ Despite this progress, due to the physiological complexity of pregnancy and the lack of large, well-characterized pregnancy and birth cohorts, our understanding of the mechanistic links between gut microbiota alterations and pregnancy complications remains limited.^10^ Publications on the gut microbiome exceeded 70,000 over the past five years, whereas only approximately 2,000 focused specifically on its association with pregnancy complications, highlighting a pronounced and persistent knowledge gap in this critical area of maternal–fetal health research (Figure 1).

This review summarizes the current understanding of how the gut microbiome is associated with various complications during pregnancy, including the underlying mechanisms and potential implications for maternal and fetal health. We focus primarily on human evidence to ensure clinical relevance while integrating mechanistic insights from animal models where necessary. We aim to highlight current gaps to guide future studies and to inform the development of microbiome-based diagnostic, preventive, and therapeutic strategies in obstetric care.

## 2. Dynamics of the maternal gut microbiome during pregnancy

During pregnancy, the maternal gut microbiome undergoes substantial remodeling from the first (T1) to the third (T3) trimester. Evidence from a longitudinal human cohort of 91 pregnant women indicates T1 gut community resembles that of healthy nonpregnant adults.^5^ This early-pregnancy microbiota is characterized by high diversity, overall stability, and an abundance of beneficial butyrate-producing taxa such as *Faecalibacterium* and *Eubacterium* (order Clostridiales). By T3, however, human gut microbial profiles exhibit a marked shift, with increased abundances of Proteobacteria and Actinobacteria (including Enterobacteriaceae and *Streptococcus*), accompanied by a pronounced reduction in microbial diversity.^5^ Experimental evidence from germ-free mice demonstrates that T3-associated microbiota induced obesity, insulin resistance, and low-grade inflammation.^5^ While these host-driven adaptations may be beneficial, as they promote energy storage in fat tissue and support fetal development.

In addition to bacterial remodeling, the maternal gut mycobiome (i.e., fungal microbiome) also undergoes significant compositional shifts during pregnancy, primarily between T1 and T2.^11^ Overall fungal diversity and richness decrease from early to late pregnancy, independent of maternal diet or pregnancy outcomes.^11^ Across stages, the relative abundance of *Saccharomyces* increases, while *Aspergillus*-dominated communities decline, and *Candida* remains relatively stable.^11^ The expansion of *Saccharomyces-*dominant enterotype is associated with enhanced gut microbial functionalities. These dynamic changes may reflect host-driven adaptations that promote metabolic efficiency and immune tolerance to support fetal development.^12^

The dynamics of other microbiomes during pregnancy, including viruses, archaea, and protozoa, remain largely unexplored. A few studies have observed a stable maternal gut virome from late pregnancy through the early postpartum period. The *α*-diversity of the viral community remained unchanged, and the overall viral composition showed no major shifts.^13^ Collectively, existing data reveal that maternal gut microbiota during pregnancy exhibits dynamic and divergent trajectories. These shifts are closely linked to adaptations in host metabolic and immune functions,^14^^,^^15^ suggesting that maternal gut microbiota play important roles in supporting pregnancy. Understanding these dynamics of the whole microbial community is thus of great biological and clinical importance, yet remains an area in urgent need of further exploration.

## 3. Gut microbiome and pregnancy complications

As discussed, the gut microbiome experiences dynamic remodeling throughout pregnancy that is essential for a healthy pregnancy. Beyond these physiological adaptations, alterations in specific microbial taxa have been increasingly associated with pregnancy complications, suggesting that microbial dysregulation may contribute to adverse maternal and fetal outcomes.^16^ Despite more extensive research in specific areas such as GDM, most studies in this field involve relatively small sample sizes and are predominantly case‒control in design, with gut microbiota analysis approach limited to 16S rRNA sequencing (Figure 1). This highlights the fact that our understanding of pregnancy complications and their microbial associations is still in its infancy and underscores the need for more comprehensive and methodologically robust research. In the following sections, we review the epidemiological evidence linking the maternal gut microbiome to pregnancy complications. We first discuss complications during gestation (Table 1), including miscarriage, excess gestational weight gain (EGWG), GDM, PE, and ICP. Subsequently, we examine perinatal and adverse birth outcomes, specifically PTB, FGR, and perinatal depression and anxiety (PDA, Table 2). Additionally, evidence regarding the gut mycobiome and virome is provided in Table 3.

**Table 1.** Summary of associations between maternal gut microbiota and pregnancy complications during gestation.

Reference	Study design	Location	N	Sequencing	Time	Identified genera and species (+: positive association, −: negative association)
**Miscarriage**						
Liu Y., et al.^17^	Case–control	China	60	16S	T1	(+) *Prevotellaceae_NK3B31_group*, *Bacteroidales_S24_7_group*, *and Eubacterium ruminantium_group* (−) *Prevotella_1*, *Prevotellaceae_UCG_003*, *Roseburia*, *Selenomonas_1*, *Gammaproteobacteria*, *Prevotellaceae*
Li Z., et al.^18^	Case–control	China	27	16S	T1	(+) *Clostridium_sensu_stricto_1_unclassified*, *Escherichia−Shigella_unclassified*, *Klebsiella_pneumoniae*, *Streptococcus_salivarius*, *uncultured_Klebsiella_sp* (−) *Megamonas_unclassified*, *Bacteroides_unclassified*, *Agathobacter_unclassified*, *Faecalibacterium_unclassified*, *Bacteroides_uniformis*
Gao B., et al.^19^	Case–control	China	100	16S	T1	(+) *Vibrio*, *Clostridium*, *Roseburia*, *Bacteroides*, *Laospirillum*, *Pseudomonas*, *Ciliates*, *Bacillus* (−) *Prevotella*, *Parabacteroides*, *Lachnospira*
Chen Y., et al.^20^	Case–control	China	30	16S	T1	(+) *Bacteroides*, *Klebsiella*, *Klebsiella_pneumoniae*, *uncultured_bacterium_g_Subdoligranulum*, *Bacteroides_uniformis*, *Dialister_sp* (−) *Escherichia*, *Subdoligranulum*, *Prevotella*, *Dialister*, *Prevotella_copri*, *Escherichia_coli*
**Gestational diabetes mellitus**						
Ma S., et al.^21^	Nested case–control	China	196	16S	T1	(+) *Eisenbergiella*, *Tyzzerella 4*, *Lachnospiraceae NK4A136 group* (−) *Parasutterella*, *Parabacteroides*, *Megasphaera*, *Dialister*, *Ruminococcaceae UCG 005*, *Ruminococcaceae UCG 002*, *Ruminococcaceae UCG 003*, *Eubacterium xylanophilum group*, *Eubacterium eligens group*
Pinto Y., et al.^22^	Prospective cohort	Israel	394	16S	T1	(−) *Prevotella*, *Prevotella copri*
Sun Z., et al.^23^	Nested case–control	China	240	Metagenome	T1, T2, T3	(+) *Bacteroides massiliensis*, *Fusobacterium mortiferum*, *Anaerostipes hadrus*, *Escherichia coli*, *Eubacterium ramulus* (−) *Bifidobacterium dentium*, *Ruminococcus bromii*, *Alistipes putredinis*, *Bacteroides ovatus*, *Clostridium* sp. CAG:58
Zhong S., et al.^24^	Nested case–control	China	93	16S	T1	(−) *Bacteroides_H*, *Acetatifactor*, *Megasphaera_A_38685*
					T2	(+) *Fusobacterium_A*, *Fournierella*, *Scatomonas* (−) *Anaerotruncus*, *Coprobacter*, *Angelakisella*
Wang S., et al.^25^	Nested case–control	China	269	Metagenome	T1, T2, T3	(+) *Fusobacterium mortiferum*, *Anaerotignum lactatifermentans*, *Ruminococcus gnavus* (−) *Akkermansia muciniphila*, *Firmicutes bacterium CAG 170*, *Cloacibacillus porcorum*, *Victivallis vadensis*
Gupta A., et al.^26^	Case–control	China Malay, India	69	16S	T3	(+) *Collinsella*, *Blautia*, *Bifidobacterium*, *Dorea*, *Roseburia*, *Coprococcus*, *Anaerostipes*, *Ruminococcus gnavus group*, *Ruminococcus torques group*, *Eubacterium hallii group*, *Romboutsia*, *Fusicatenibacter*, *Clostridium sensu stricto 1*, *Agathobacter*, *Ruminococcus*, *Megasphaera* (−) *Akkermensia*, *Bacteroides*, *Acidaminococcus*, *Escherichia-Shigella*, *Klebsiella*, *Lachnospiraceae NK4A136 group*
Gao Y., et al.^27^	Prospective cohort	China	30	16S	T1	(+) *Bifidobacterium*, *Methanobrevibacter* (−) *Dialister*
					T3	(+) *Bifidobacterium*, *Ezakiella* (−) *Victivallis*, *Turicibacter*
Wu X., et al.^28^	Case–control	China	104	16S	T1	(+) *Prevotella 9*, *Sutterella*, *Odoribacter* (−) *Ruminococcaceae UCG-002*, *Phascolarctobacterium*, *Prevotellaceae NK3B31 group*, *Methanobrevibacter*
					T2	(+) *Sutterella*, *Ruminococcus gnavus group*, *Erysipelatoclostridium* (−) *Megamonas*, *Eubacterium eligens group*, *Butyrivibrio*, *Paraprevotella*, *Fusobacterium*
					T3	(+) *Sutterella* (-) *Erysipelotrichaceae UCG-003*, *Paraprevotella*, *Fusobacterium*, *Coprococcus*
Yao W., et al.^29^	Case–control	China	61	16S	T1	(+) *Escherichia-Shigella*, *Finegoldia*, *Klebsiella* (−) *Bacteroides*, *Prevotella*, *Bifidobacterium*, *Faecalibacterium*, *Akkermansia*
**Excess gestational weight gain**						
Kennedy K.M., et al.^30^	Prospective	Germany	65	16S	T1, T2, T3, Postpartum	(+) *Bifidobacterium* (−) *Lachnospiraceae NK4A136 group*, *Ruminococcaceae UCG-002*, *Ruminococcaceae UCG-014*, *Ruminococcaceae UCG-010*, *Ruminococcaceae UCG-005*, *Coprococcus 1*
Preeclampsia						
Chen X., et al.^31^	Case–control	China	152	16S	T3	(+) *Clostridium*, *Dialister*, *Fusobacterium*, *Veillonella* (−) *Faecalibacterium*, *Akkermansia*, *Lachnospira*
Chang Y., et al.^32^	Case–control	China	63	16S	T3	(+) *Blautia*, *Eubacteriumrectale*, *Eubacteriumhallii*, *Streptococcus*, *Subdoligranulum* (−) *Bifidobacterium*, *Collinsella*, *Alistipes*
Wang J., et al.^33^	Case–control	China	50	16S	T3	(−) unidentified *Lachnospiraceae*
Lv L.-J., et al.^34^	Case–control	China	77	Metagenome	T3	(+) *Pauljensenia bouchesdurhonensis*, *Ruminococcus gnavus* (−) *Akkermansia muciniphila*, *Bilophila wadsworthia*, *Prevotella bivia*, *Paraprevotella clara*, *Barnesiella intestinihominis*
Meijer S., et al.^35^	Case–control	Sweden	54	16S	T3	(−) *Collinsella*, *Akkermansia*, *Cloacibacillus*
Liu X., et al.^36^	Case–control	China	18	Metagenome	Postpartum	(−) *Limosilactobacillus fermentum*
Li G., et al.^37^	Case–control	China	54	16S	T3	(+) *Clostridium methylpentosum* (−) *Faecalibacterium prausnitzii*
Zhao Y., et al.^38^	Case–control	China	86	16S	T3	(+) *Shigella*, *Fusobacterium*, *Streptococcus* (−) *Bacteroides*, *Ruminococcus*, *Oscillospira*
**Intrahepatic cholestasis in pregnancy**						
Li G-H., et al.^39^	Case–control	China	30	16S	T3	(+) *Parabacteroides*, *Bilophila*, *Bacteroides*, *Escherichia/Shigella* (−) *Faecalibactium*, *Bifidobacterium*, *Blautia*
Li R., et al.^40^	Case–control	China	58	16S	T3	(+) *Blautia*, *Citrobacter*, *Streptococcus*, *Streptococcus luteciae*
Zhan Q., et al.^41^	Case–control	China	90	16S	T3	(+) *Flavonifractor*, *Atopobium*, *Turicibacter*, *Parabacteroides*, *Lactobacillus*, *Escherichia_Shigella*, *Olsenella* (−) *Megamonas*

**Table 2.** Summary of associations between maternal gut microbiota and perinatal pregnancy complications.

Reference	Study design	Location	N	Sequencing	Time	Identified genera and species (+: positive association, −: negative association)
**Preterm birth**						
Miao Z., et al.^42^	Prospective cohort	China	5,313	16S, Metagenome	T1, T2	(+) *Burkholderia-Caballeronia-Paraburkholderia*, *Bacteroides*, *Ralstonia*, *Parabacteroides*, *Lachnoclostridium*, *Odoribacter*, *Anaerostipes*, *Clostridium innocuum* (−) *Solobacterium*, *Holdemania*, *Ruminociccus gaureauii group*
Yu H.-R., et al.^43^	Nested case–control	China	211	16S	T2	(+) *Actinomyces* spp.
Yin C., et al.^44^	Case–control	China	33	16S	T2	(+) *Fusobacterium*, *Streptococcus*, *Neisseria*, *Haemophilus*, *Lautropia*, *Porphyromonas*, *Clostridium*, *Prevotella*, *Rothia*, *Oscillospira*, *Granuliccatella*, *Actinomyces*, *Bilophila* (−) *Coprococcus*, *Gemmiger*
**Fetal growth restriction**						
Tang H., et al.^45^	Case–control	China	20	16S	Postpartum	(+) *Bacteroides*, *Akkermansia*, *Eubacterium_coprostanoligenes_group*, *Phascolarctobacterium*, *Parasutterella*, *Odoribacter*, *Lachnospiraceae_UCG_010*, *Dielma* (−) *Dialister*, *Tyzzerella*, *Collinsella*, *Roseburia*, *Intestinibacter*, *Monoglobus*, *Clostridium_sensu_stricto_1*, *Veillonella*, *Corynebacterium*, *Anaerococcus*, *Staphylococcus*, *Eubacterium*, *DTU089*, *Eubacterium_brachy_group*
Kosinska-Kaczynska, K., et al.^46^	Case–control	Poland	22	Metagenome	T3	(+) *Veillonella*, *Limosilactobacillus*, *Bacteroides*, *Arabiibacter*, *Oxalobacter*
Tu X., et al.^47^	Case–control	China	32	16S	Postpartum	(+) *Bacteroides*, *Faecalibacterium Lachnospira*
Xiao Y., et al.^48^	Case–control	China	35	16S	T3	(+) *Bacteroides* (−) *Escherichia-Shigella*, *Bifidobacterium*
Ruebel M.L.,^49^	Nested case-control	Guatemala	265	16S	T1	(+) *Barnesiella*, *Faecalibacterium*, *Sutterella*, *Odoribacter*, *Hafnia*, *Bacterioides*
					T3	(+) *Bacteriodes*, *Flavonifractor*, *Acinetobacter*, *Fusobacterium* (−) *Megasphaera*, *Phascolarctobacterium*, *Turicibacter*
Yu H.-R., et al.^50^	Nested case–control	China	217	16S	T2	(+) *Dorea* spp., *Bacteroides ovatus* (−) *Rosenburia faecis*
Tao Z., et al.^51^	Case–control	China	70	16S	T3	(+) *Lactobacillus*, *Catenibacterium* (-) *Ruminococcaceae*, *Bacteroides uniformis*, *Mollicutes RF39*, *Alistipes onderdonkii*
He X., et al.^52^	Case–control	China	16	Metagenome	T3	(+) *Marinisporobacter*, *Sphingomonas*, *Ornatilinea*, *Plesiomonas* (−) *Roseburia*, *Dysgonomonas*, *Anaerovibrio*, *Mobilisporobacter*, *Thermohydrogenium*, *Cellulosimicrobium*, *Roseomonas*, *Aquabacterium*
**Perinatal depression and anxiety**						
Hieta J., et al.^53^	Prospective cohort	Finland	419	Metagenome	T1	(+) *Clostridium* sp. AF27-2AA, *GGB3571 SGB4778*, *Streptococcus parasanguinis*, **Streptococcus* salivarius*
					T3	(+) *Bacteroides clarus*, *Bacteroides faecis*, *Bacteroides xylanisolvens*, *Hungatella hathewayi*, *Lachnospira SGB5076*, *Streptococcus thermophilus*, *Hydrogeniiclostidium mannosilyticum*, *Bacteroides xylanisolvens*, *Clostridiales bacterium Choco116*, *Flavonifractor plautii*, *Intestinimonas butyriciproducens*, *Sellimonas intestinalis*
Xu S., et al.^54^	Prospective cohort	China	196	16S	T1, T2, T3, Postpartum	(+) *Erysipelotrichaceae_UCG-003*, *Streptococcus*, *Anaerostipes* (−) *Lachnospiraceae_UCG-001*, *Dialister*, *Cloacibacillus*
Chi R., et al.^55^	Prospective cohort	China	87	Metagenome	T1, T2, T3,	(+) *Oscillibacter* sp. KLE 1745, *Oscillibacter* sp. PEA192, *Oscillibacter* sp. KLE 1728, *Oscillospiraceae bacterium VE202 24*, *Treponema socranskii*
Matsunaga M., et al.^56^	Case–control	Japan	339	16S	Postpartum	(+) *Escherichia–Shigella* (−) *Lachnospira*, *Veillonella*, *Alistipes*, *Phascolarctobacterium*
Xie T., et al.^57^	Case–control	China	86	16S	T3	(+) *unclassified_f_Lachnospiraceae*, *Lachnospiraceae_UCG-001*, *Butyricicoccus*, *unclassified_c_Clostridia*, *unclassified_f_Enterobacteriaceae*, *Enterobacter*, *unclassified_k_norank_d_Bacteria* (−) *Family_XIII_UCG-001*, *F0332, Anaerococcus*, *Ezakiella*, *Fenollaria*, *Peptoniphilus*, *Candidatus_Soleaferrea*, *Porphyromonas*, *norank_f_norank_o_Oscillospirales*, *Enterococcus*, *norank_f_Eggerthellaceae*, *Enterorhabdus*, *TM7x*

**Table 3.** Summary of associations between maternal gut mycobiome, virome and pregnancy complications.

Reference	Study design	Location	N	Sequencing	Time	Identified taxa (+: positive association, −: negative association)
**Gestational diabetes mellitus**						
Wu X., et al.^13^	Nested case–control	China	102	Metagenome	T1	(+) *Escherichia phage SH2026Stx1*, *Enterobacteria phage mEp043 c-1* *crAssphage cr50_1*, *Enterobacteria phage phi80*, *Escherichia phage HK106* (−) *Eubacterium eligens*, *Escherichia phage HK106*
Ferrocino I., et al.^58^	Case–control	Italy	162	26S	T2	(+) Ascomycota, *Kluyveromyces*, *Metschnikowia*, *Pichia* (−) Basidiomycota, *Saccharomyces*, *Clavispora*, *Cystobasidium*
Fu Y., et al.^11^	Nested case–control	China	1059	ITS2	T1, T2, T3	(+) *Mucor*
Wu N., et al.^20^	Case–control	China	49	ITS1	T2	(+) *Hanseniaspora*, *Torulaspora*, *Kazachstania*, *Trichoderma*, *Pichia*, *Acremonium*, *Sticta*, *Cladophialophora*, *Lophiostoma*, *Gibberella* (−) *Basidiomycota*, *Ganoderma*, *Volvariella*, *Marasmius*, *Tricholoma*, *Rhizomucor*
**Preeclampsia**						
Meijer S., et al.^35^	Case–control	Sweden	54	ITS1	T3	(−) *Peniophora*
Zou H., et al.^59^	Case–control	China	12	ITS1, ITS2	T3	(+) Ascomycota, Sordariomycetes, Glomerellales
Lv L.-J., et al.^60^	Case–control	China	77	Metagenome	T3	(+) Inoviridae

### 3.1. Miscarriage

Miscarriage, defined as the loss of a pregnancy before fetal viability, occurs in approximately 15% of clinically recognized pregnancies.^61^ Great efforts have been made to analyze the vaginal and endometrial microbiome, with studies showing that miscarriage is associated with reduced abundance of *Lactobacillus* spp. at T1.^62^^,^^63^ However, evidence regarding the gut microbiome in this context remains scarce. Women who experienced miscarriage exhibited a distinct gut microbiota composition profile, characterized by reduced *α*-diversity and an increased Firmicutes/Bacteroidetes ratio.^17^^,^^19^^,^^20^ At the genus level, *Bacteroides* were reported to be enriched in cases, and *Prevotella* tended to be more abundant in healthy controls.^17^^,^^19^^,^^20^ Although a growing body of literature has begun to address the role of the maternal gut microbiome in miscarriage, most research to date has been restricted to the analysis of bacterial composition. Furthermore, recurrent miscarriage, a clinically challenging subtype of pregnancy loss, remains underexplored.

### 3.2. Gestational diabetes mellitus

GDM is a condition characterized by hyperglycemia first identified during pregnancy, and affects nearly 14% of pregnancies globally.^64^ Research in this area is relatively extensive, encompassing not only bacterial taxa but also emerging evidence on fungal and viral communities. Distinct microbial patterns have also been observed as early as T1, prior to clinical diagnosis of GDM. Women who later develop GDM typically show reduced gut microbial richness and diversity, an elevated Firmicutes/Bacteroidetes ratio,^21^^,^^24^^,^^25^ and lower relative abundances of genera such as *Prevotella* and *Sutterella.*^22^^,^^28^ At the species level, GDM is associated with enrichment of *Fusobacterium mortiferum*, and depletion of beneficial species such as *Akkermansia muciniphila* and *Alistipes putredinis.*^23^^,^^25^^,^^29^ These patterns resemble those observed in obesity and type 2 diabetes, reflecting pro-inflammatory microbial signatures and insulin-resistant metabolic states.^65^ By T3, GDM is also characterized by increased abundances of *Ruminococcus* and *Bacteroides*, together with persistent reductions in beneficial taxa such as *Akkermansia* and *Faecalibacterium.*^23^^,^^26^ Species-level changes, including enrichment of *Ruminococcus obeum* and depletion of *Dialister* sp. CAG:357, have been consistently reported.^25,66–69^ Longitudinal studies further demonstrate that these dynamic microbial shifts may contribute to abnormal maternal glucose and lipid metabolism, highlighting the key role of gut microbiota in GDM progression.^27^^,^^70^

Alternations in the T1 fungal community were also implicated in GDM development. Overall, GDM patients showed a distinct mycobiota composition at T1, with increased *α*-diversity compared to healthy controls.^58^ Specifically, a higher abundance of the genus *Mucor* was associated with a higher risk of GDM.^11^ In T2, the mycobiome in GDM patients demonstrated increased abundances of *Kluyveromyces*, *Pichia,* and *Metschnikowia*, and reduced *Saccharomyces*, *Clavispora*, and *Cystobasidium* levels.^58^ Similarly, alterations were also described in the gut virome of GDM patients. While diversity analysis revealed no difference in the overall gut virome composition,^13^ the abundances of several phages, including *Escherichia phage SH2026Stx1*, *Enterobacteria phage mEp043 c-1*, *crAssphage cr50_1*, *Enterobacteria phage phi80,* and *Escherichia phage HK106* were significantly different at T1 between GDM patients and healthy control.^13^ To sum up, studies examining the relationship between gut microbiota and GDM have consistently reported an enrichment of dysbiotic microbial profiles associated with systemic inflammation and metabolic disturbances. Research focusing on early pregnancy has demonstrated that microbial signatures, together with clinical indicators, can be incorporated into predictive models for GDM with promising accuracy.^71^ Nevertheless, investigations at the species level remain scarce, highlighting the need for further research to fully unravel the mechanisms underlying the interaction between gut microbiota and GDM.

### 3.3. Excess gestational weight gain

EGWG is defined as weight gain during pregnancy that exceeds recommended guidelines.^72^ Both EGWG and maternal obesity have been consistently associated with a higher risk of pregnancy complications and long-term metabolic dysfunction in both the mother and their offspring.^53^^,^^73^^,^^74^ Observational studies have reported reduced gut microbial *α*-diversity among overweight women during pregnancy.^75^ Beyond overall diversity, EGWG has been linked to specific taxonomic signatures. At the genus level, EGWG is characterized by depletion of several short-chain fatty acid (SCFA)-producing genera, including *Ruminococcaceae UCG-005*, and a gut barrier-associated *Lachnospiraceae NK4A136 group.*^30^^,^^76^ Conversely, increased abundances of *Blautia*, *Bifidobacterium,* and *Bacteroidetes* have been associated with EGWG.^75^^,^^77^^,^^78^ At the species level, a negative association with *Akkermansia muciniphila* and a positive association with *Escherichia coli* have been reported in women with EGWG.^77^ Interestingly, prior pregnancies may have lasting impacts on maternal microbial adaptation and gestational weight gain in subsequent pregnancies, highlighting the need for longitudinal sampling to capture these dynamic changes.^30^

Emerging evidence also indicates that maternal adiposity influences the gut mycobiome. For instance, women with pre-pregnancy overweight exhibit greater temporal variability in fungal community composition across pregnancy trimesters compared with underweight women,^11^ suggesting altered fungal dynamics in response to metabolic perturbations. Research on the maternal gut virome in relation to obesity and EGWG remains limited, representing an important knowledge gap in understanding the full spectrum of microbiome–metabolism interactions during pregnancy.

### 3.4. Preeclampsia

PE is a form of hypertensive disorder of pregnancy,^79^ characterized by the onset of hypertension and significant proteinuria after 20 weeks of gestation, and can progress to eclampsia or multiorgan failure.^80^ Microbial composition analyses have consistently demonstrated shifts according to the PE status. At the phylum level, women with PE exhibit an increased relative abundance of Bacteroidetes and a decreased abundance of Firmicutes.^33^^,^^35^^,^^37^^,^^81^ This depletion is also observed in beneficial genera such as *Faecalibacterium*, *Akkermansia*, *Bifidobacterium*, *Oscillibacter*, and *Cloacibacillus.*^31^^,^^32^^,^^35^^,^^38^^,^^82^ At the species level, *Limosilactobacillus fermentum* is enriched in women with PE during late pregnancy,^36^ whereas healthy controls have higher levels of *Alistipes putredinis*, *Bacteroides vulgatus*, *Ruminococcus torques*, *Akkermansia muciniphila,* and *Clostridium citroniae.*^34^^,^^83^

In addition, fungal and viral alterations have been reported, with increased abundance of the *Ascomycota* phylum in the mycobiome and the Inoviridae family in the virome of women with PE.^59^^,^^60^ Despite these compositional changes, most studies found no significant differences in the overall diversity of the bacteriome, mycobiome, or virome.^38^^,^^59^^,^^60^ Therefore, prospective studies and mechanistic investigations are warranted to identify early-pregnancy microbial biomarkers that could enhance prediction and prevention of PE.

### 3.5. Intrahepatic cholestasis of pregnancy

ICP is the most common pregnancy-specific liver disease, clinically diagnosed by gestational pruritus accompanied by elevated serum bile acid level,^84^ and affects approximately 0.1–2% of pregnant women worldwide.^85^ Gut microbiome profiling in women with ICP is characterized by a reduced relative abundance of Firmicutes and enrichment of Bacteroidetes.^39^^,^^86^ Genus-level depletion is consistently observed in beneficial taxa, particularly SCFA producers such as *Faecalibacterium*, *Eubacterium hallii group*, and *Megamonas.*^39^^,^^40^ In contrast, the ICP-associated microbiome is enriched in genera involved in bile acid transformation, including *Citrobacter*, *Streptococcus*, *Parabacteroides*, and *Bilophila*, as well as potentially opportunistic pathogens such as *Escherichia_Shigella.*^40^^,^^41^ At the species level, *Bacteroides fragilis* has been identified as a key microbial signature of the ICP gut microbiota,^87^ consistent with its established role in bile acid metabolism and host–microbe immune interactions. However, research on other microbial domains, such as the gut mycobiome and virome in ICP, is still very limited. Integrative multikingdom studies are therefore needed to comprehensively understand the role of the gut ecosystem in ICP.

### 3.6. Preterm birth

PTB, defined as birth before 37 weeks of gestation, is the leading cause of neonatal and under-five mortality worldwide.^88^ The majority of population-based research on PTB has traditionally focused on the gut microbiota trajectories of newborns or the association between maternal vaginal microbiota and PTB.^89^ In contrast, large cohort studies exploring the relationship between maternal gut microbiota and PTB have only begun to emerge and gradually increase in the past few years.^90^

As early as T1, the gut microbiota can indicate the risk of PTB. In T1, genera such as *Burkholderia-Caballeronia-Paraburkholderia*, *Bacteroides*, *Ralstonia*, *Parabacteroides*, *Lachnoclostridium*, *Odoribacter*, and *Anaerostipes* show a positive association with PTB, while genera such as *Solobacterium*, *Holdemania*, *Ruminociccus gaureauii group* exhibit a negative association. Additionally, *Clostridium innocuum* has been identified as a species associated with PTB, potentially influencing parturition by affecting maternal estradiol and progesterone levels.^42^ Postpartum studies have also shown that mothers who experienced PTB are characterized by a reduction in *α*-diversity. In the PTB group, *Clostridiales*, *Bifidobacterium*, and *Streptococcus* are significantly reduced, while *Lactobacillales* are markedly increased.^91^^,^^92^ While the fungal genus *Mucor* has been positively associated with PTB,^11^the underlying mechanisms are still unknown. This knowledge gap extends to the gut virome, where epidemiological evidence remains limited.

### 3.7. Fetal growth restriction

FGR, also known as intrauterine growth restriction, is a pathological condition in which a fetus fails to achieve its expected growth potential.^93^ Evidence based primarily on 16S rRNA sequencing has shown that FGR is associated with reduced maternal gut microbial *α*-diversity in T1, while no significant differences in *β*-diversity are observed throughout gestation.^49^ In T1, the relative abundances of genera *Barnesiella*, *Faecalibacterium*, *Sutterella*, *Odoribacter*, *Hafnia*, and *Bacteroides* are increased in women with FGR.^48^^,^^49^ In T2, species such as *Dorea* spp. and *Bacteroides ovatus* are increased, whereas *Rosenburia faecis* is decreased.^50^ During T3, the abundances of gut *Lactobacillus* and *Catenibacterium* are increased in women with FGR,^51^ while *Sphingomonas* and *Marinisporobacter* are negatively correlated with neonatal body mass index and birth weight.^52^ Overall, these taxa associated with FGR are largely implicated in nutrient absorption and maternal energy metabolism.^94^ However, research investigating the influence of early pregnancy gut microbiota on embryonic development remains limited, and the roles of maternal gut virome and mycobiome in FGR are still unknown. Further research in these areas is needed.

### 3.8. Perinatal depression and anxiety

Maternal mental disorders, particularly PDA, are among the most common morbidities during pregnancy and the postnatal period.^95^ These conditions can have adverse effects not only on the mother's well-being, but also on her child and family.^96^ PDA is often assessed by the self-rating anxiety scale and depression scale.^54^^,^^55^ Recent evidence suggests that maternal depression and anxiety during pregnancy are associated with reduced microbial diversity, a decrease in SCFA-producing genera (e.g., *Faecalibacterium*, *Bifidobacterium*, and *Blautia*), and an increase in proinflammatory taxa (e.g., *Escherichia*–*Shigella*, *Eggerthella*, and *Parabacteroides*).^55-57^^,^^97^ These alterations have been associated with perinatal mood-related outcomes, and are commonly discussed within the conceptual framework of the gut–brain–placenta axis.^98^ However, longitudinal human data across trimesters are still lacking, particularly integrative analyzes of the gut mycobiome and virome. These gaps underscore the need for multiomic and mechanistic studies in maternal mental health.

In summary, emerging evidence from both human and animal studies suggests that the maternal gut microbiome plays an active role in regulating pregnancy progression, fetal development, and maternal physiological adaptation. However, despite growing interest, current findings remain inconsistent across studies. The variability in sampling timepoints, sequencing platforms, and bioinformatic pipelines contributes to this lack of reproducibility. Furthermore, associations identified at higher taxonomic levels often obscure the distinct or even opposing functions of microbes at the species or strain level. These challenges highlight the need for more refined, high-resolution investigations and mechanistic studies to elucidate causal pathways and identify reliable microbial biomarkers for the early prediction, prevention, and treatment of pregnancy complications.

## 4. Mechanisms underlying the association between gut microbiome and pregnancy complications

The gut microbiota and its derived metabolites are recognized as key regulators of maternal physiology. By encoding a vast repertoire of enzymatic proteins and metabolic pathways absent in the host genome, the gut microbiome significantly expands the host's functional capacity, acting as a virtual endocrine and metabolic organ.^99^ This microbial-host crosstalk is critical in modulating nutrient metabolisms, regulating hormonal signaling, and shaping immune tolerance.^100^ Dysbiosis or disruption of these microbial functions has been increasingly linked to pregnancy complications. A deeper understanding of how the gut microbiota influences these processes may provide novel mechanistic insights into both normal gestational physiology and the etiology of pregnancy-related disorders.

### 4.1. Metabolic response

#### 4.1.1. Short-chain fatty acids

**Figure 2.**
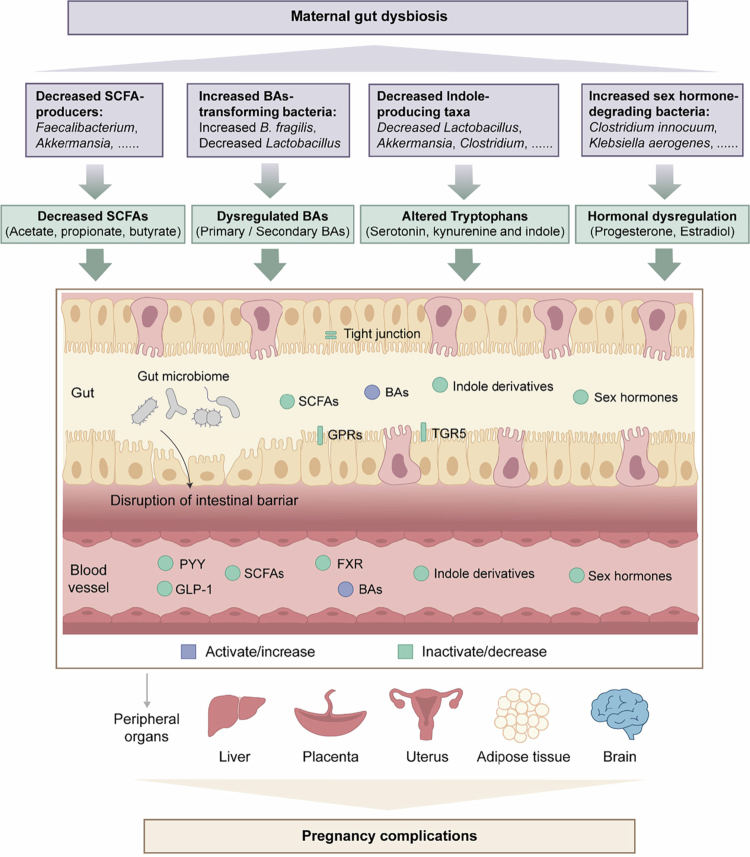
Metabolomic pathways linking gut dysbiosis to pregnancy complications. This diagram illustrates four major mechanisms through which maternal gut microbiome dysbiosis contributes to pregnancy complications. SCFAs metabolism: Beneficial bacteria produce SCFAs (acetate, propionate, butyrate) through dietary fiber fermentation. These metabolites regulate glucose and lipid homeostasis via GPRs such as GPR41 and GPR43, facilitate the release of GLP‐1 and PYY from enteroendocrine L‐cells, modulate blood pressure through renin regulation, and influence neurodevelopment by crossing the blood‒brain barrier. Reduced SCFAs-producing bacteria result in organic metabolic dysregulation and placental endothelial dysfunction, leading to pregnancy complications. Bile acid metabolism: Dysbiosis alters the transformation of primary bile acids into secondary bile acids. These molecules act as signaling ligands for receptors such as TGR5 and FXR, influencing systemic metabolism and placental function. Imbalanced bile acid profiles are linked to hepatic and placental pathologies during pregnancy. Tryptophan metabolism: Alterations in tryptophan-metabolizing taxa affect the production of indole derivatives, serotonin, and kynurenine. Indole derivatives play a crucial role in maintaining the intestinal barrier integrity and modulating the inflammation at the maternal–fetal interface, thus contributing to immune disequilibrium and fetal resorption. Hormonal modulation: Gut bacteria possessing steroid-metabolizing enzymes can degrade sex hormones, particularly estradiol and progesterone. Alterations in these microbial activities affect uterine, brain, and placenta, contributing to pregnancy complications. Abbreviations: SCFAs: short-chain fatty acids; BAs: bile acids; GPRs, G protein-coupled receptors; PYY, peptide tyrosine tyrosine; GLP-1, glucagon‐like peptide‐1; FXR: farnesoid X receptor; TGR5, Takeda G protein-coupled receptor 5.

SCFAs, primarily acetate, propionate, and butyrate, are key microbial metabolites generated through dietary fiber fermentation and serve as central regulators of maternal metabolism, immune homeostasis, and vascular function during pregnancy (Figure 2).^101^

In GDM, decreased abundances of SCFA-producing genera such as *Faecalibacterium*, *Roseburia*, and *Akkermansia* have been consistently observed, accompanied by lower circulating or fecal SCFA levels.^102^ Mechanistically, SCFAs modulate host glucose and lipid metabolism by binding to G protein–coupled receptors GPR41 and GPR43.^103^ Activation of these receptors on enteroendocrine L cells induces the secretion of glucagon-like peptide-1 (GLP-1) and peptide YY (PYY), which enhance insulin secretion and improve glucose homeostasis.^104^^,^^105^ At the tissue level, acetate and butyrate support hepatic lipogenesis and glycogen synthesis, while propionate acts as a substrate for gluconeogenesis and suppresses fatty acid synthesis via inhibition of fatty acid synthase.^106^ In adipose tissue, SCFAs activate GPR43 to suppress lipolysis, reduce circulating free fatty acids, and promote adipogenesis through peroxisome proliferator-activated receptor *γ* (PPARγ) signaling and induction of fasting-induced adipose factor (FiAF).^107^^,^^108^ In skeletal muscle and liver, SCFAs enhance AMP-activated protein kinase (AMPK) and PPARγ coactivator-1α (PGC-1α) signaling, thereby increasing fatty acid oxidation and energy expenditure.^109^^,^^110^ Collectively, reduced SCFA levels in GDM disrupt these metabolic regulatory pathways, leading to impaired insulin sensitivity, altered lipid metabolism, and dysregulated glucose homeostasis, which together contribute to the pathophysiology of GDM. Notably, these lipid- and adipose-related pathways overlap substantially with mechanisms implicated in maternal obesity and excessive gestational weight gain, suggesting shared microbiota–metabolic axes across pregnancy-related metabolic disorders.^109^

In PE, several studies have reported declined fecal acetate and butyrate concentrations, along with depletion of *Mitsuokella*, *Clostridium Leptum*, *Akkermansia muciniphila,* and *Oscillibacter*, taxa known for SCFA production.^111^^,^^112^ SCFAs act on GPCR such as GPR41, GPR43, and olfactory receptor 78 (Olfr78) expressed in vascular smooth muscle, renal tissue, and immune cells, thereby modulating vascular tone and immune homeostasis.^113^^,^^114^ For example, propionate binding to GPR41 reduces blood pressure via renin suppression, whereas activation of Olfr78 by acetate or propionate promotes renin release, maintaining dynamic blood pressure balance.^113^ In parallel, SCFAs enhance autophagy in placental macrophages and promote their anti-inflammatory macrophage autophagy, M2 polarization, and trophoblast invasion via activation of AMPK‐mTOR (mammalian target of rapamycin), GPR43‐STAT1 (signal transducer and activator of transcription 1), and GPR41‐AKT (protein kinase B) signaling pathways.^82^ Consequently, SCFA deficiency may exacerbate endothelial dysfunction, inflammation, and placental maladaptation, forming a mechanistic link between gut dysbiosis and the development of PE.

In women experiencing PDA or high stress, the intestinal microbiota tends to show reduced abundance of SCFA-producing bacteria such as *Lachnospira*, *Veillonella*, *Alistipes*, and *Phascolarctobacterium.*^57^ Mechanistically, SCFAs act as endogenous ligands for orphan GPCRs, and intracellular SCFAs affect gene expression by inhibiting histone deacetylases (HDACs), the activity of which was implicated in brain development and various neuropsychiatric disorders, including depression.^115^ SCFAs are capable of crossing the blood-brain barrier and strengthening its integrity by regulating the expression of tight junction proteins.^116^^,^^117^ Moreover, SCFAs modulate the hypothalamic–pituitary–adrenal (HPA) axis by attenuating corticosterone secretion and restoring glucocorticoid receptor sensitivity, thereby dampening the stress response.^118^^,^^119^ Consequently, reduced SCFA levels during pregnancy may heighten vulnerability to mood dysregulation by amplifying inflammation and impairing neuroendocrine balance.

Furthermore, emerging data indicate that both mothers carrying growth-restricted fetuses and the infants themselves exhibit reduced levels of gut butyrate-producing bacteria.^94^ This reduction may impair placental nutrient transport, angiogenesis, and mucosal homeostasis.^52^

#### 4.1.2. Bile acids

Bile acids play a crucial role in facilitating fat digestion and absorption in the small intestine. Beyond digestion, they act as key signaling molecules that regulate lipid and glucose metabolism, inflammation, and energy expenditure.^120^ Primary bile acids, cholic acid and chenodeoxycholic acid, are synthesized in the liver and conjugated before entering the intestine, where gut microbiota such as *Bacteroides* and *Lactobacillus*, reshape the bile acid pool through deconjugation, oxidation, and 7-dehydroxylation (Figure 2).^121^

This host–microbiota bile acid axis is critically involved in pregnancy-related metabolic and hepatic disorders.^87^ In ICP mouse models, the enrichment of *Bacteroides fragilis* drives excessive bile acid deconjugation. This species exhibits high bile salt hydrolase activity, leading to a bile acid profile that fails to adequately activate farnesoid X receptor (FXR) signaling. The resulting bile acid accumulation promotes hepatic inflammation and cholestatic injury.^122^

Dysregulated bile acid signaling also has metabolic consequences relevant to GDM. Specific conjugated and secondary bile acids, including taurocholic acid and taurolithocholic acid, are positively associated with impaired glucose tolerance.^123^ Through FXR- and Takeda G protein-coupled receptor 5 (TGR5)-dependent pathways, altered bile acid profiles influence hepatic gluconeogenesis, insulin sensitivity, and incretin secretion, linking gut microbial bile acid metabolism to gestational glucose dysregulation.^124^

#### 4.1.3. Tryptophan metabolites

The gut microbiome plays a role in the three major tryptophan metabolism pathways, including serotonin, kynurenine, and indole derivatives, all implicated in a wide spectrum of human diseases.^125^ While the serotonin pathway regulates gastrointestinal signaling and the kynurenine pathway serves as a central hub for immune tolerance,^126^^,^^127^ microbial-derived indole metabolites represent a critical interface between gut microbes, vascular health, and immune regulation during pregnancy.^128^ Key taxa such as *Lactobacillus*, *Akkermansia*, and *Clostridium* species metabolize tryptophan into indole derivatives, including indole-3-propionic acid (IPA).^129^ These metabolites exert antioxidant and anti-inflammatory effects, preserve endothelial integrity, and protect against oxidative stress (Figure 2).^129^

In PE, reduced circulating IPA has been linked to endothelial dysfunction and placental maladaptation, highlighting the protective role of microbial indole signaling in pregnancy-associated vascular diseases.^82^^,^^130^ Altered tryptophan metabolism may also contribute to GDM pathophysiology. Indole derivatives enhance intestinal barrier integrity and suppress lipopolysaccharide (LPS) translocation, thereby limiting low-grade inflammation that drives insulin resistance.^131–135^

#### 4.1.4. Hormonal regulation

During pregnancy, a complex interplay develops between the gut microbiota and sex hormones. Experimental studies have also demonstrated that gut microbes possess the ability to convert steroids into sex hormones and regulate levels of estradiol, progesterone, and androgens, indicating their modulatory role in pregnancy health (Figure 2).^136-138^

Microbial degradation of estradiol and progesterone has been implicated in PTB. In particular, *Clostridium innocuum*, capable of degrading both hormones, is associated with higher PTB risk.^42^ Mechanistically, depletion of estradiol and progesterone triggers preterm parturition by disrupting myometrial quiescence and placental vascular integrity.^139^^,^^140^ Progesterone deficiency leads to functional withdrawal with upregulation of contraction-associated proteins, while reduced estradiol impairs placental angiogenesis and favors a pro-inflammatory, contractile uterine environment,^141^ suggesting a gut–hormone axis that influences the onset of PTB.

Beyond obstetric outcomes, estradiol–microbiota interactions also influence maternal mental health.^142^ In women with PDA, altered gut microbiota and reduced estrogen levels frequently co-occur.^56^^,^^143^ Experimental colonization with estradiol-degrading bacteria such as *Klebsiella aerogenes*, a gut bacterium, degrades estradiol via 3β-hydroxysteroid dehydrogenase (3β-HSD), lowers circulating estradiol, and induces depression-like behaviors in female mice, providing causal evidence for microbial modulation of hormonal and affective pathways.^144^

### 4.2. Intestinal barrier permeability

The intestinal barrier, composed of the mucus layer, epithelial cells, and mucosal immune defenses, functions as a critical gatekeeper that prevents the translocation of luminal microbes and their products into the systemic circulation.^145^ During normal pregnancy, this barrier undergoes adaptive remodeling to accommodate metabolic and immunological demands.^146^ However, increases in intestinal permeability, often referred to as “leaky gut”, have emerged as a key mechanistic link connecting gut dysbiosis to pregnancy-associated systemic inflammation.^145^^,^^147^

Intestinal barrier integrity is tightly regulated by microbial-derived metabolites and host hormonal signals discussed above. Microbial products such as bacterial toxins, LPS, or bile acids and SCFAs generated through bacterial deconjugation and dehydroxylation play a central role in maintaining epithelial defense and barrier function.^148^ For instance, SCFAs, particularly butyrate, are essential for maintaining the epithelial barrier by linking the intracellular energy sensor and upregulating the expression of tight junction proteins such as zonula occludens-1 (ZO-1), occludin, and claudins.^149^ In parallel, enrichment of bile acid–modifying bacterial species can disrupt bile acid homeostasis. Altered bile acid profiles independently influence epithelial permeability through receptor-mediated and detergent-like effects on the intestinal epithelium.^145^^,^^150-152^ In addition to microbial metabolites, estrogens contribute to barrier maintenance by supporting epithelial structural integrity and regulating intestinal permeability.^150^ Moreover, specific pathobionts, including *Enterococcus gallinarum*, *Proteus mirabilis*, and *Escherichia coli,* can translocate across the intestinal epithelial barrier, disseminate to extraintestinal tissues, and promote systemic inflammation and disease.^153-155^

When this barrier is compromised, microbial products, most notably LPS from gram-negative bacteria, translocate into the portal and systemic circulation, initiating a state of metabolic endotoxemia.^156^^,^^157^ Consistently, increased intestinal permeability and elevated circulating LPS levels have been reported in women with recurrent miscarriage.^158^ In the context of EGWG and gestational obesity, increased serum zonulin, indicating higher intestinal permeability, is associated with metabolic risk markers.^159^ Similarly, an increased lactulose/mannitol ratio, a hallmark of leaky gut, has been observed in ICP patients.^160^ Beyond these conditions, early-pregnancy markers of intestinal barrier dysfunction, including soluble CD14 (sCD14) and lipopolysaccharide-binding protein (LBP), have been linked to PTB in *Plasmodium falciparum*–infected pregnancies.^161^ Through facilitating the systemic dissemination of microbial products, leaky gut serves as a critical conduit by which localized gut dysbiosis is translated into maternal immune activation and inflammation.^162^

### 4.3. Immune response

Pregnancy presents a unique immunological challenge, as the maternal immune system must tolerate the semi-allogeneic fetus while protecting the mother.^163^ Across gestation, immune activity follows a regulated trajectory: T1 is largely pro-inflammatory, T2 is anti-inflammatory, and T3 reverts to a pro-inflammatory state.^164^ Disruptions to this balance can result in excessive inflammation, impaired placental function, and loss of fetal tolerance, contributing to pregnancy complications.^165^

**Figure 3.**
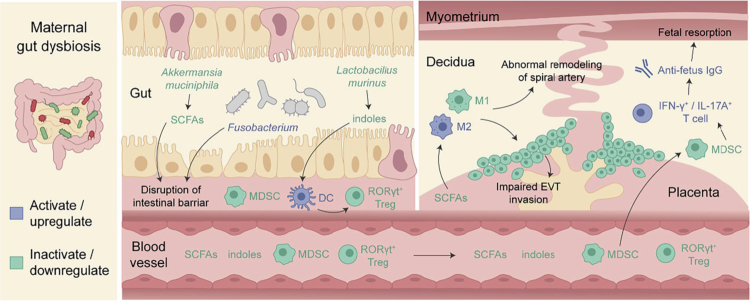
Gut microbiota-driven immune regulation at the maternal–fetal interface. This schematic illustrates how maternal gut microbiota dysbiosis undermines immune tolerance at the maternal–fetal interface through integrated metabolic and immunological mechanisms, leading to adverse pregnancy outcomes. Reduction in beneficial gut bacteria, such as *Akkermansia muciniphila* and *Lactobacillus murinus*, results in markedly decreased production of microbiota-derived metabolites, including SCFAs and indole derivatives. The consequent reduction in circulating SCFAs limits their availability to the placenta and initiates a cascade of pathogenic events, including maternal hypertension, inhibition of autophagy in placental macrophages, skewing of macrophage polarization from an anti-inflammatory M2 phenotype toward a proinflammatory M1 phenotype, and impaired EVT invasion. These alterations collectively contribute to abnormal placental and embryonic development, as well as defective spiral artery remodeling. Concurrently, an increased abundance of pathogenic bacteria, particularly *Fusobacterium*, together with compromised intestinal barrier integrity, facilitates bacterial translocation from the gut to the placenta. This process exacerbates placental inflammation and further disrupts placental growth and function. In parallel, dysbiosis-associated depletion of microbiota-dependent tryptophan metabolites weakens immune tolerance at the MFI by promoting antigen-presenting cells such as DC, impairing the maintenance of MDSCs and gut-derived RORγt⁺ Tregs. The loss of these immunoregulatory pathways permits the expansion of IFN-γ⁺ and IL-17⁺ effector T cells, promoting a proinflammatory milieu that upregulates anti-fetus IgG and favors fetal resorption and pregnancy failure. Abbreviations: SCFAs, short-chain fatty acids; EVT, extravillous trophoblast; dNK, decidual natural killer cell; DC, dendritic cell; Treg, regulatory T cell; MDSC, myeloid-derived suppressor cell; IgG, immunoglobulin G.

Fetal tolerance is actively established and maintained at the maternal-fetal interface (MFI) through coordinated regulation of innate and adaptive immune cells.^166-170^ Increasing evidence indicates that this immune equilibrium is shaped by upstream signals derived from the maternal gut microbiota.^166^ Microbiota-derived metabolites, such as SCFAs and tryptophan derivatives, as well as pregnancy-associated hormones, fine-tune this cellular network and define the inflammatory “set-point” at the MFI.^166^^,^^170^^,^^171^ Decidual macrophages and dendritic cells integrate microbial signals to modulate antigen presentation and cytokine production.^163^^,^^172^ Microbial metabolites also promote tolerance by priming myeloid-derived suppressor cells (MDSCs) and RORγt^+^ regulatory T cells (Tregs).^170^^,^^173^ Tregs restrain T helper cells 17 (Th17) in the uterus, where controlled interleukin-17 (IL-17) supports placentation and extravillous trophoblast (EVT) invasion.^174-176^ Disruption of Th17/Treg balance leads to increased anti-fetus IgG, fetal resorption, abnormal remodeling of spiral arteries, and impaired embryonic and placental development, ultimately contributing to implantation failure, preeclampsia, and recurrent miscarriage (Figure 3).^177-179^

Consequently, the disruption of this microbiota-driven immune regulation underpins the pathogenesis of various pregnancy complications. In recurrent miscarriage, gut dysbiosis is characterized by an increased Firmicutes/Bacteroidetes ratio, indicative of a pro-inflammatory microbial profile.^17^ This dysbiosis drives a systemic inflammatory state characterized by activated cytotoxic CD8⁺ T cells, NK cells, and mucosal-associated invariant T (MAIT) cells,^180^ accompanied by elevated endometrial expression of Nalp-3, caspase-1, and IL-1β, IL-2, IL-17A, IL-17F, tumor necrosis factor-*α* (TNF-*α*), and interferon-*γ* (IFN-*γ*).^17^^,^^158^ Experimental evidence also showed that administration of indole-3-carbinol or colonization with tryptophan-metabolizing *Lactobacillus murinus* rebalances the Th17/Treg response at the MFI and prevents fetal resorption in germ-free mice.^170^

Similarly, women who develop GDM exhibit gut microbial dysbiosis, characterized by decreased anti-inflammatory taxa, including *Akkermansia*, *Bifidobacterium,* and increased pro-inflammatory taxa, including Enterobacteriaceae.^29^ These alterations are associated with elevated circulating inflammatory markers, including IL-6, TNF-*α*, and C-reactive protein (CRP).^181^ In a study using germ-free mice, transplantation of T1 fecal microbiota from women who later developed GDM induced higher insulin resistance, accompanied by decreased Tregs and increased pro-inflammatory Th17 responses,^5^^,^^182^ highlighting a microbiota-driven immune mechanism underlying metabolic dysfunction in GDM.

In PE, maternal gut dysbiosis-induced disruption of the gut barrier facilitates LPS translocation, activating Toll-like receptor 4 (TLR4) signaling and downstream p38 MAPK pathways.^183^ This induces placental inflammation, impaired trophoblast invasion, defective spiral artery remodeling, and endothelial dysfunction.^31^^,^^82^^,^^184^^,^^185^ Furthermore, PE-associated gut microbiota induces Treg/Th17 imbalance, oxidative stress, and vascular injury in germ-free pregnant animals, supporting a causal role of microbiota-driven immune dysregulation in PE pathogenesis.^31^ Single-cell analyzes further reveal that these immune breakdowns manifest as impaired NK cell function and altered HLA-F signaling at the MFI.^186^

PTB is associated with reduced abundances of anti-inflammatory gut microbes, including *Bifidobacterium*, *Clostridium*, and *Bacteroides,*^91^ which are known to promote Treg differentiation and IL-10 production.^187^^,^^188^ For instance, *Bifidobacterium* strains inhibit LPS-induced nuclear factor kappa B (NF-κB) activation, as well as the production of IL-8 and cyclooxygenase-2 (COX-2) through activation of intercellular adhesion molecule 1 (ICAM-1).^187^ This inflammatory milieu drives leukocyte infiltration into the decidua and myometrium, where they release matrix metalloproteinases (MMPs) that degrade the extracellular matrix of the fetal membranes and cervical stroma.^189^^,^^190^ Consequently, these pro-inflammatory cytokines, particularly IL-1β and TNF-*α*, synergistically upregulate the expression of contraction-associated proteins and stimulate prostaglandin synthesis, effectively transitioning the uterus from a quiescent to a contractile state to initiate preterm parturition.^187^^,^^191^^,^^192^

Accumulating evidence proposes that systemic inflammation plays a central role in linking gut dysbiosis to PDA. Fecal microbiota transplantation from women with PDA into animal models induces depressive-like behaviors, accompanied by increased plasma LPS levels, activation of the NLRP3–NF-κB signaling pathway, and elevated expression of TNF-*α* and IL-6.^193^^,^^194^ This neuroinflammatory state disrupts neurotransmitter metabolism and suppresses brain-derived neurotrophic factor (BDNF) signaling, thereby affecting monoaminergic and glutamatergic pathways involved in mood regulation.^56^ These findings support a causal role of gut microbiota–driven immune activation in the development of prenatal mood disorders.

Beyond bacterial influence, the role of other microbial kingdoms in early-life immune programming remains complex. Currently, no evidence links fungal mycobiota changes to immune tolerance within the placental decidua. However, emerging studies highlight the contribution of the gut virome to early-life immune programming. Specifically, infant gut virome show a higher abundance of active temperate phages compared to maternal virome, although their role during pregnancy remains to be fully elucidated.^195^^,^^196^

## 5. Beyond pregnancy: maternal gut microbiome shapes offspring health

Beyond complications throughout gestation, there is an increasing recognition that the maternal gut microbiome influences not only maternal health but also the long-term health and development of the offspring.^197^^,^^198^ Preclinical studies have suggested two key routes by which the maternal microbiome influences offspring health. First, microbial metabolites can cross the placental barrier, exerting effects both before and after birth.^167^^,^^199^^,^^200^ Second, microbes themselves are vertically transmitted to the infant during vaginal delivery, and later through breastfeeding and skin contact.^201-203^ These pathways collectively seed the neonatal gut microbiota, forming the foundation for immune and metabolic development.^204^

Early studies relied on cultivation or genus/species-level taxonomic profiling, which often failed to distinguish shared species from true vertical transmission.^205-207^ Recent strain-level metagenomic and meta-transcriptomic approaches have revolutionized this field by allowing precise detection of maternal-origin strains and assessing their transcriptional activity in infants.^208^ Moreover, horizontal gene transfer (HGT) has emerged as an additional mechanism through which maternal microbes can pass functional genes, especially those related to carbohydrate and amino acid metabolism, to the neonatal gut.^209^ Together, these findings reveal a complex, multi-layered process by which maternal microbes colonize and shape the infant gut.

Notably, while maternal skin- and vaginal-derived strains colonize the infant gut only transiently, maternally derived gut strains show markedly greater persistence and ecological fitness than microbes acquired from other sources.^204^ Therefore, pregnancy complications such as GDM, PE, and PTB are often accompanied by maternal intestinal dysbiosis. A significant disruption in microbial transmission could profoundly alter the early microbial composition and functional landscape of the offspring's gut. In mouse models, maternal intestinal *Akkermansia muciniphila* was demonstrated to alter offspring neuronal and intestinal stem-cell proliferation by modulating circulating SCFAs and amino acids, thus enhancing mTOR signaling in fetal stem cells.^210^ Besides, maternal gut bacteria altered by IL‑17A‑driven inflammation reshape the chromatin landscape of offspring CD4⁺ T cells, increasing their susceptibility to intestinal inflammation and neurodevelopmental disorders such as autism spectrum disorders.^211^ Similarly, infants of mothers with PTB harbored distinct *Bacteroides* and *Escherichia coli* profiles, with reduced carbohydrate metabolic capacity.^204^ In women with PDA, a decreased abundance of maternal gut bacteria involved in immune modulation and neurotransmitter regulation is associated with impaired infant microbiome establishment and delayed neuroimmune maturation.^212-214^

**Figure 4.**
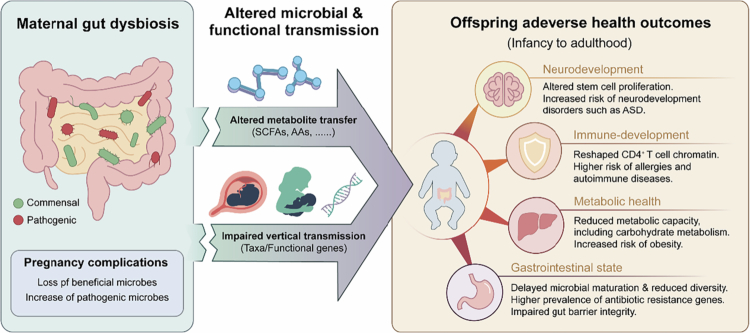
Maternal-to-offspring microbiome transmission and long-term health implications. This schematic depicts the multiple routes of maternal microbiome transmission to offspring and the consequences of maternal gut dysbiosis. Maternal gut microbes and their metabolites influence fetal development through two primary pathways: (1) Metabolite transfer: Microbial metabolites, including SCFAs, amino acids, and neuroactive compounds, cross the placental barrier during pregnancy. (2) Vertical microbial transmission: Maternal microbial taxa colonize the infant gut through vaginal delivery, and breastfeeding, as well as functional genes transmission through horizontal gene transfer. Pregnancy complications disrupt both metabolite profiles and microbial transmission fidelity, leading to altered neonatal gut microbiome composition and function. These early-life perturbations have lasting consequences, increasing offspring risk for metabolic disorders (obesity, diabetes), immune dysfunction (allergies, autoimmune diseases), neurodevelopmental disorders (behavioral problems, cognitive impairment), and gastrointestinal dysfunctions (altered microbiota, antibiotic genes) that extend into childhood and adulthood. The diagram emphasizes the critical window of early microbial colonization in shaping lifelong health trajectories. Abbreviations: SCFAs, short-chain fatty acids; AAs, amino acids; ASD, autism spectrum disorder.

The developmental trajectory of the gut microbiome during infancy can exert long-lasting effects on host growth, immunity, and neurodevelopment. Early colonizers play a pivotal role in educating the immune system, shaping gut barrier integrity, and regulating host metabolism.^215^^,^^216^ Perturbations in microbial succession, whether from cesarean delivery, antibiotic exposure, or altered maternal transmission, are linked to increased risks of obesity, allergies, autoimmune diseases, and neurodevelopmental disorders in later life.^217^^,^^218^ For instance, delayed microbial maturation in infants delivered by cesarean section is linked to lower microbial diversity and a higher prevalence of antibiotic resistance genes.^219^ Likewise, early-life gut dysbiosis has been found to predict an increased risk of later metabolic dysfunction and susceptibility to inflammatory diseases in later childhood and beyond.^220^^,^^221^ For example, increased expression of microbial histidine ammonia lyase in the infant gut microbiome can lower histidine levels and alter urocanate/histidine and urocanate/glutamate ratios in the perirhinal cortex, thereby influencing the gut–brain communication and neurodevelopment.^222^ Interestingly, experimental evidence reported a single commensal strain that corrects oxytocin levels and synaptic potentiation can prevent social deficits in maternal obese mouse offspring.^223^ Collectively, these data underscore that the fidelity and timing of maternal microbial transmission are critical determinants of long-term offspring health, linking prenatal and perinatal microbial ecology to disease risk across the lifespan (Figure 4).

## 6. Conclusions and future perspectives

The maternal gut microbiome holds considerable promise as a novel source of predictive biomarkers for pregnancy complications. While most studies to date have focused on taxonomic shifts at high levels (e.g., phylum, genus, or species), strain-level signatures, functional gene profiles, and microbial-derived metabolites may offer greater specificity and mechanistic insights for early risk stratification for pregnancy complications like GDM, PE, PTB, FGR, and maternal mental disorders. To advance the field, future research could leverage artificial intelligence-integrated multiomics data, including microbial metagenome, host metabolome, and genome, etc., to identify robust, strain-level biomarkers for early risk stratification.

An important limitation across the existing literature on maternal pregnancy disorders is the insufficient control and characterization of maternal diet, which represents a major upstream determinant of both the gut microbiome and circulating metabolites. Dietary intake may profoundly shape microbial composition, functional capacity, and metabolite production, and therefore could confound or modify microbiome–pregnancy outcome associations.^224^^,^^225^ Future studies should incorporate standardized dietary assessments, longitudinal dietary tracking, or controlled feeding designs, and explicitly model diet–microbiome interactions when investigating pregnancy complications. Integrating high-resolution dietary data with multiomics approaches will be essential to disentangle diet-driven microbial variation from disease-specific microbial signatures and to enable more precise, mechanism-informed interventions targeting maternal nutrition and metabolic health.

**Figure 5.**
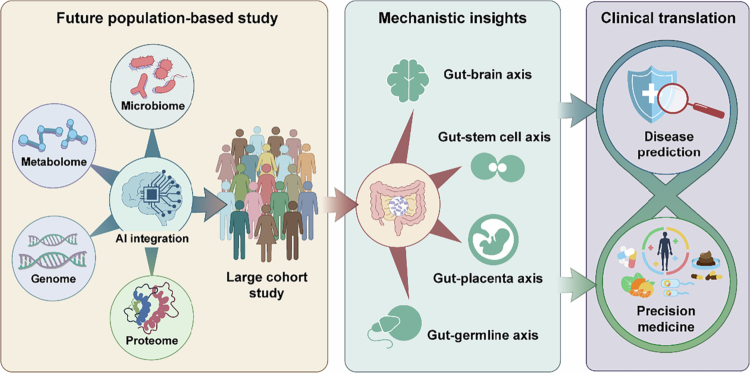
Future directions in maternal gut microbiome research. Future directions encompass integration of multiomic approaches (metagenomics, metabolomics, transcriptomics) to identify strain-level and functional signatures for early prediction of pregnancy complications; mechanistic research of human cohorts with experimental models to elucidate causal pathways, including the gut‒brain axis, gut‒germline axis, gut‒placenta axis, gut-stem cell axis, etc.; and biomarker development and therapeutic interventions. Development of early-pregnancy predictive biomarkers for disease prediction, as well as clinical assessment of targeted strategies including probiotics, prebiotics, dietary modifications, postbiotics, and rationally designed live biotherapeutic products, with rigorous safety evaluation in pregnant populations.

Furthermore, moving beyond association to causation requires rigorous mechanistic research combining longitudinal human cohorts with experimental models. This comprehensive understanding is a prerequisite for developing targeted therapeutic interventions aimed at mitigating pregnancy complications. As therapeutic targets, the microbiome also offers multiple intervention modalities. Broad-spectrum antibiotics should be limited to clear infectious indications. Probiotics and defined microbial consortia represent a safer, more targeted strategy, yet benefits are highly strain-specific, and many trials are underpowered.^226^ Prebiotics and dietary interventions are attractive due to favorable safety profiles, though rigorous randomized trials are still sparse.^227^ Emerging options, including postbiotics, rationally designed live biotherapeutic products, and phage-based approaches, also offer precise targeting but require thorough safety evaluation in pregnant populations (Figure 5).

In summary, the gut microbiome is a compelling target for both the prediction, prevention, and intervention of pregnancy complications, but responsible translation demands high-quality longitudinal evidence, standardized measurement, mechanistic understanding, and careful safety evaluation before routine clinical implementation.
